# Surgical Management of Interstitial Ectopic Pregnancy Using Intramyometrial Vasopressin and Cornuostomy

**DOI:** 10.7759/cureus.77125

**Published:** 2025-01-08

**Authors:** Amanjot Kaur, Pradip K Saha, Manisha Meena, Sangeeta Tvinwal

**Affiliations:** 1 Obstetrics and Gynaecology, Postgraduate Institute of Medical Education and Research, Chandigarh, IND

**Keywords:** angular pregnancy, cornual ectopic pregnancy, eccentric location, ectopic pregnancy, interstitial pregnancy, radiological diagosis of intestitial pregnancy, ruptured ectopic pregnancy

## Abstract

Interstitial pregnancy is a rare entity with a high risk of maternal mortality. It is difficult to diagnose in early pregnancy and can result in uterine rupture at the cornual end leading to catastrophic heamorrhage. The key to its management lies in early diagnosis by detailed history history, early radiological diagnosis, and management by medical or surgical means as indicated. Surgical management forms the mainstay of management in case of ruptured interstitial pregnancy or if the gestational age is advanced and the HCG levels are high. There is an inclination towards laproscopic management rather than laparotomy, and more conservative techniques involving cornuostomy rather than cornuectomy. Caution should be exercised in the next pregnancy as there is a risk of repeat intestitial pregnancy in the next conception. An elective cesarean section should be done in the next pregnancy to avoid the risk of uterine rupture at the cornuostomy site.

## Introduction

Interstitial ectopic pregnancy is an ectopic gestation that develops in the uterine part of the fallopian tube. It constitutes only 5% of all tubal ectopic pregnancies but is associated with a mortality rate of 2% to 2.5% in comparison to an overall mortality rate of 0.14% for ectopic pregnancies [1-2]. Previous ectopic pregnancy, previous ipsilateral salpingectomy, and IVF are some of the predisposing factors for such implantations. Not only are these pregnancies difficult to diagnose due to their unique location, but they are also difficult to manage as they can result in torrential bleeding in case of rupture. Here, we describe a case of interstitial live ectopic pregnancy which was suspected on ultrasonography and operated by laparotomy and cornuostomy.

## Case presentation

A 26-year-old third gravida with a history of a previous cesarean delivery and one medically managed ectopic pregnancy presented with amenorrhoea of seven weeks and five days with an ultrasound report suggestive of unruptured ectopic pregnancy. The patient was asymptomatic and the ectopic pregnancy was diagnosed on a routine first trimester scan done for localization of the pregnancy and to confirm fetal viability. On admission, the vitals of the patient were stable, and she was well-oriented to time, place, and person. Her abdomen was soft on palpation, the per speculum examination revealed a healthy-looking cervix and vagina, and no mass or cervical motion tenderness was observed on a vaginal examination.

**Figure 1:**
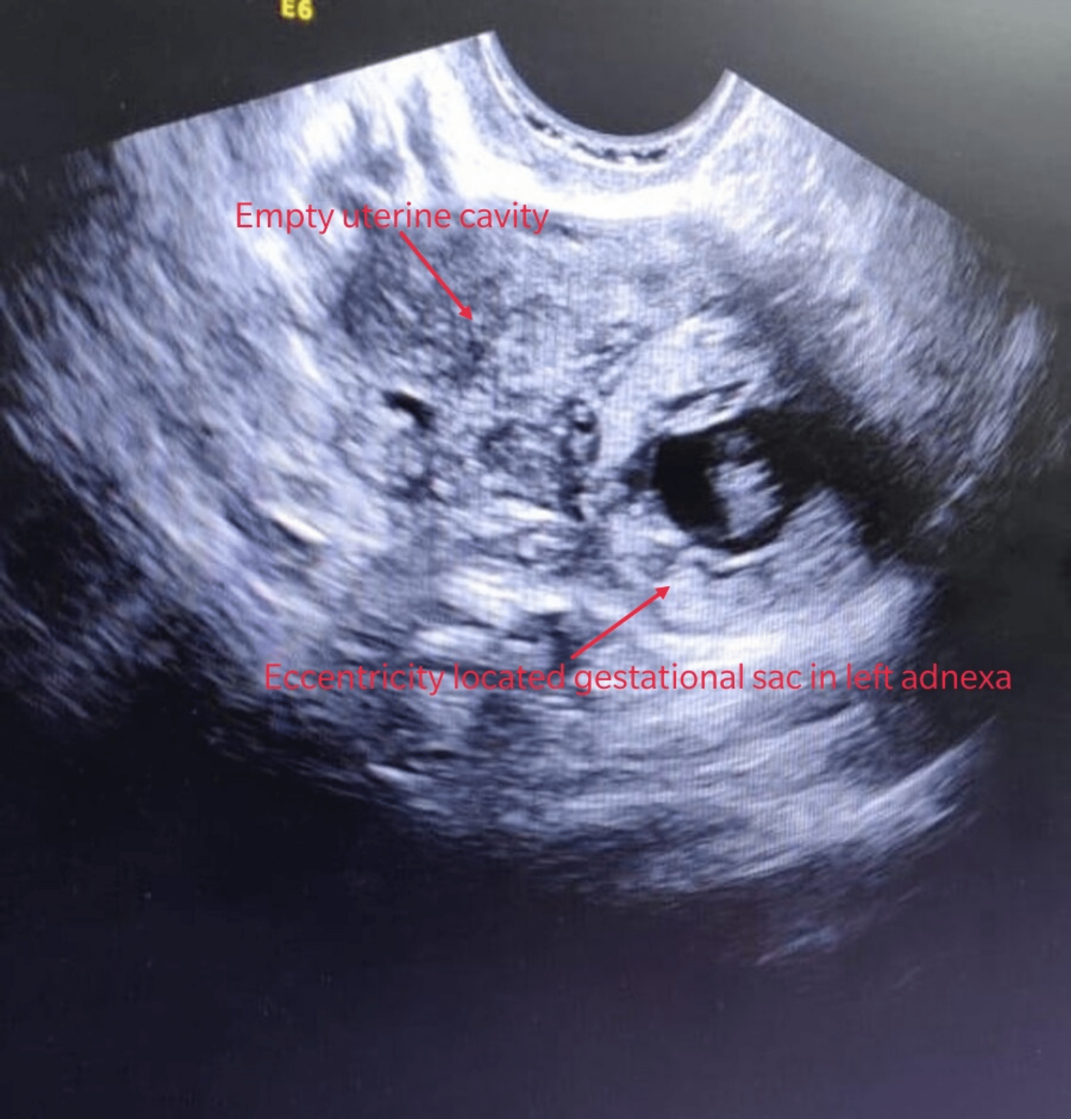
Ultrasound picture showing an empty uterine cavity and an eccentrically located gestational sac in the left adnexa

The ultrasound done at our facility was suggestive of an empty uterus and a live, eccentrically located pregnancy in the left adnexa, with a thin myometrial layer surrounding the sac. The crown rump length of the pregnancy corresponded to seven weeks (Figure 1). There was no free fluid in the pelvis and the abdomen. The HCG levels were 41000 mIU/ml.

**Figure 2:**
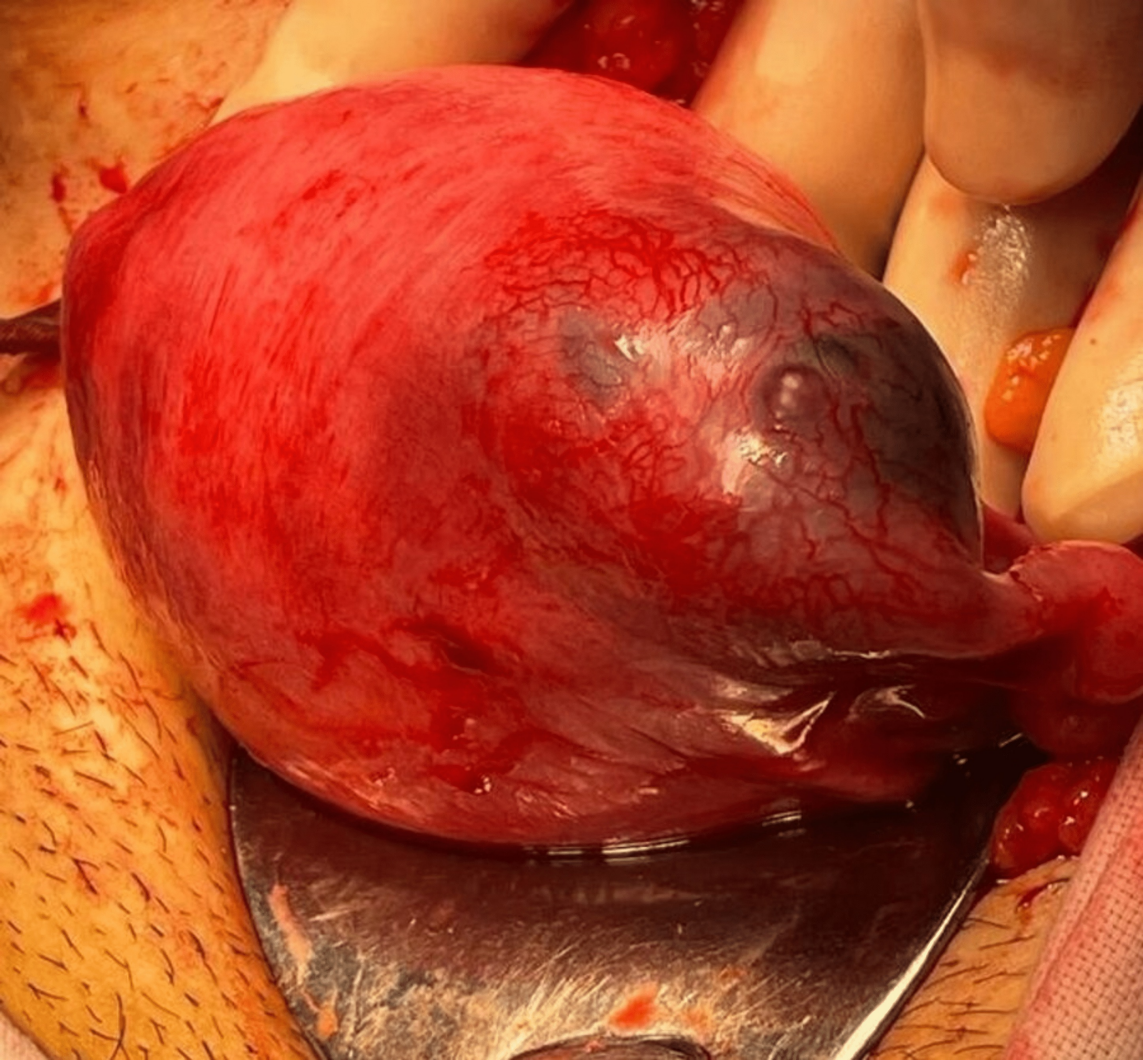
Left interstitial pregnancy as seen on opening the peritoneal cavity

**Figure 3:**
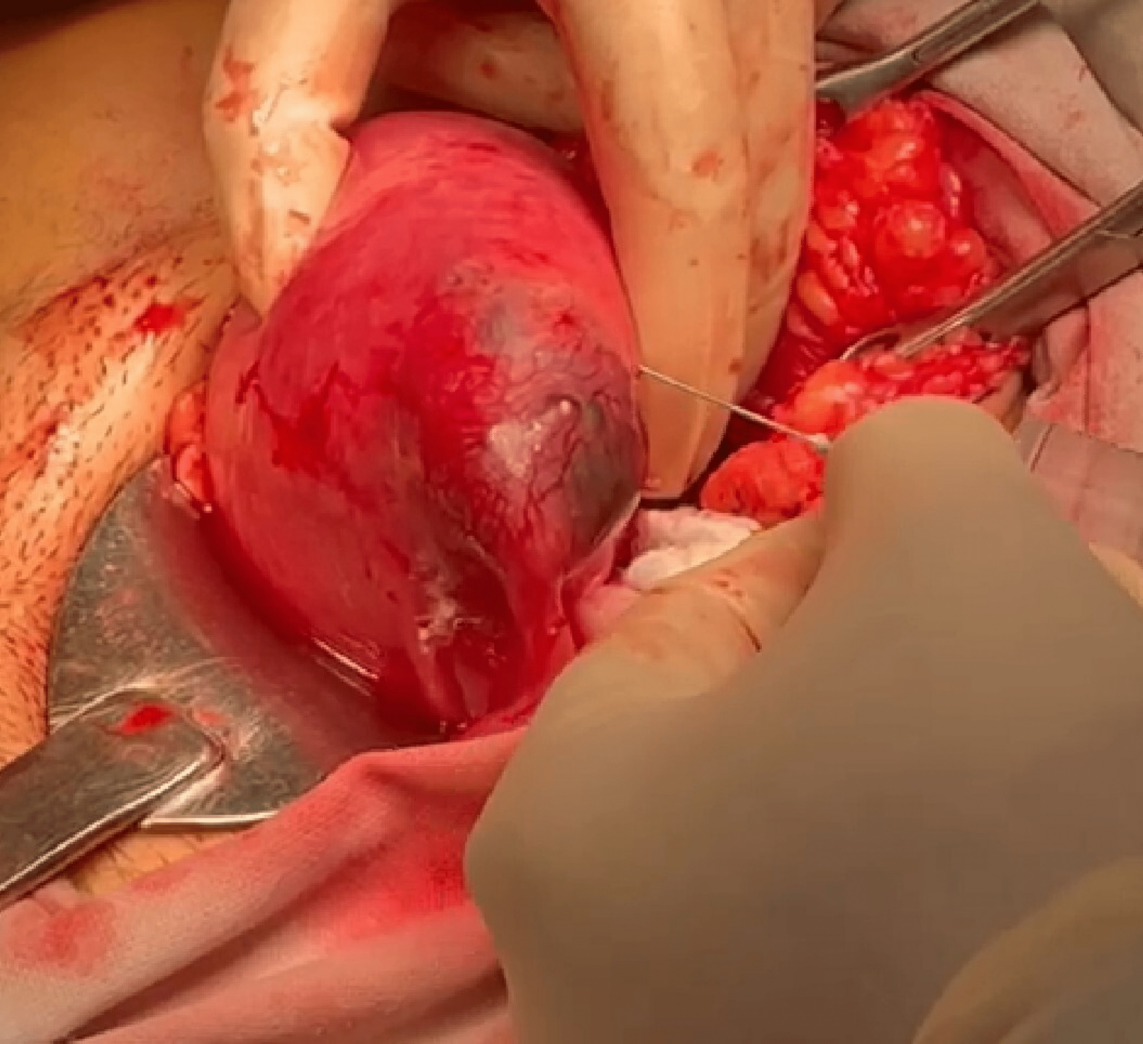
Injecting vasopressin in the myometrium surrounding the ectopic gestational sac prior to performing cornuostomy

**Figure 4:**
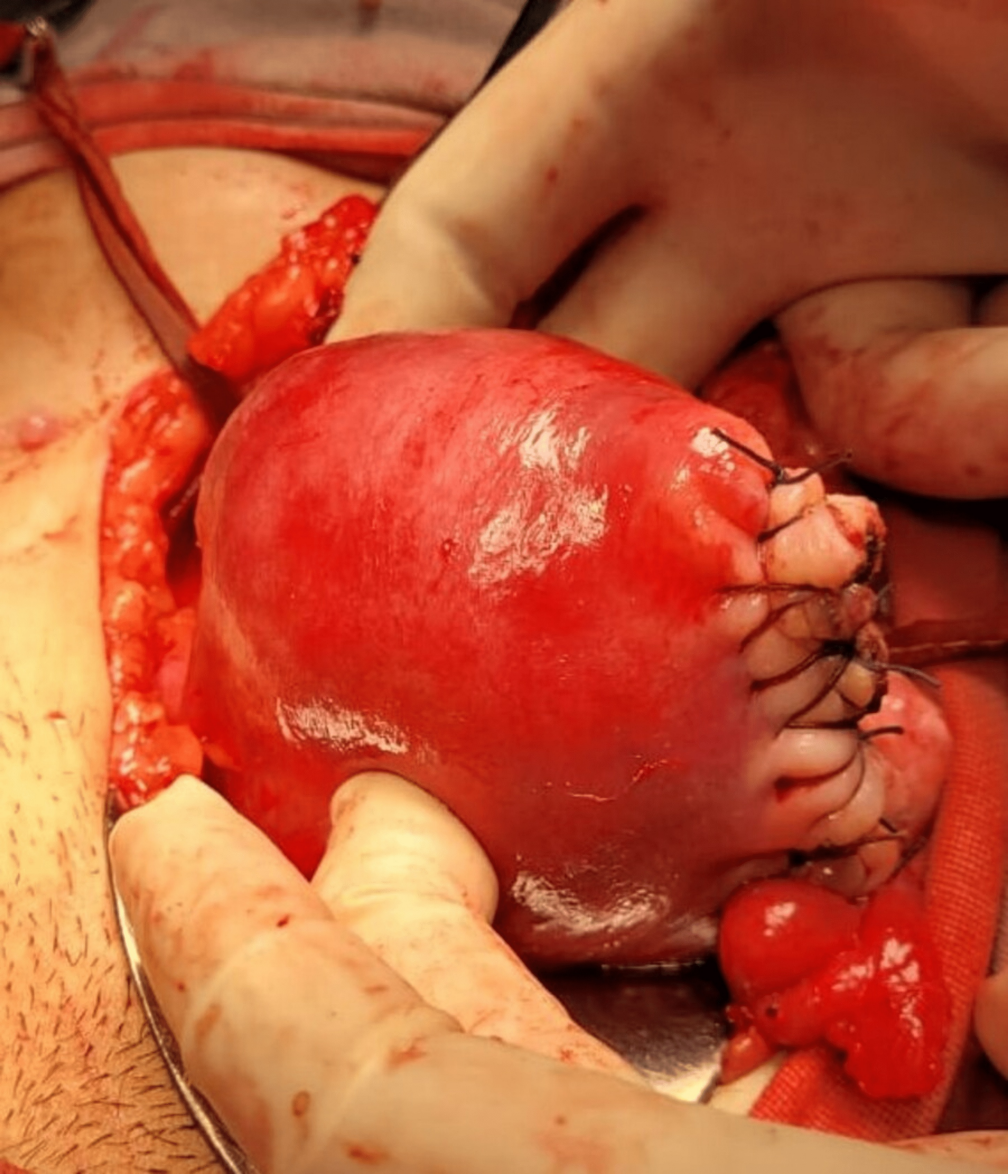
Postoperative image after performing a cornuostomy and suturing the defect with Vicryl 1.0

A decision for surgery was taken anticipating a cornual ectopic pregnancy given an eccentrically located live ectopic pregnancy and high HCG levels. Since laparoscopic facilities were not available in the emergency hours, a laparotomy was performed. Intraoperatively, the ectopic gestational sac was found to be located at the left cornua (Figure 2). A stay suture was applied at the cornual end and the myometrium around the pregnancy was infiltrated with diluted vasopressin (Figure 3). A linear incision was made over the left cornua in line with the fallopian tube and the contents of the gestational sac were evacuated. The uterine cavity was not opened while performing the cornuostomy leading to a conclusion of an interstitial pregnancy. The cornua was repaired with interrupted sutures with Vicryl 1.0 (Figure 4). The blood loss was less than 50 ml.

## Discussion

Interstitial pregnancies are rare and the risk factors include previous ectopic pregnancy, previous ipsilateral salpingectomy, and artificial reproductive techniques. Our patient had a spontaneous conception with a history of previous ectopic pregnancy that was medically managed. Given its unique location, it is difficult to diagnose and must be differentiated from cornual ectopic pregnancy. The ultrasonographic criteria for diagnosis are an empty uterine cavity, a gestational sac located eccentrically and 1 cm from the most lateral wall of the uterine cavity, and a thin (5 mm) myometrial layer surrounding the gestational sac. The “interstitial line sign” that extends from the upper region of the uterine horn to border the intramural portion of the fallopian tube has also been used. Figure 1 shows an empty uterine cavity and an eccentrically located gestational sac. Magnetic resonance imaging (MRI) and 3-dimensional ultrasonography help in better diagnosis.

There is no consensus on a standard surgical technique for the management of this entity. Conventional management of this condition has been surgical and includes cornual resection by laparotomy or laparoscopy and/or hysterectomy [3]. Recent literature reports more conservative techniques like cornuostomy rather than cornual resection or hysterectomy [4-10]. There is also a trend towards laparoscopic management rather than laparotomy. Subsequent pregnancy after a cornuostomy is a cause for concern given the risk of uterine rupture due to weakening of the cornual area. Su et al reported a uterine rupture at the site of a prior laparoscopic cornuostomy scar after a full-term vaginal delivery [9]. Cornuostomy was performed for our patient, and a laparotomy was planned instead of a laparoscopic approach given the non-availability of the same in emergency hours.

Use of diluted vasopressin, applying stay sutures, purse string sutures at the base of the mass, use of electrosurgical instruments like ENDOLOOP™, and clipping the uterine artery before proceeding to cornuostomy have been described in the literature to reduce bleeding at the cornual area [10-12]. We used a stay suture and dilute vasopressin to achieve this effect.

It is pragmatic to look for pregnancy in the same location early in gestation in the next pregnancy and to deliver the next pregnancy by an elective cesarean section to avoid the risk of uterine rupture.

## Conclusions

Interstitial pregnancies are rare, difficult to diagnose, and can be life-threatening. A conservative approach using cornuostomy instead of cornuectomy is increasingly employed for its management, in conjunction with various methods to reduce blood loss during surgery. Care should be exercised in the next pregnancy to diagnose pregnancy at the same site and to plan delivery by an elective cesarean section to avoid rupture at the cornuostomy site.
